# Supplementary material for: Exploring motivated reasoning in polarization over the unfolding 2023 judicial reform in Israel
Source: Commun Psychol. 2024 Jun 18;2:59. doi: 10.1038/s44271-024-00080-x (PMC11332199; doi:10.1038/s44271-024-00080-x)
Supplement: Supplementary file 1 — Supplementary Information [file 44271_2024_80_MOESM1_ESM.pdf]

Supplementary Online Information for:

Exploring Motivated Reasoning in Polarization over the Unfolding 2023 Judicial Reform in Israel

Author Information

Dora Simunovic[1,2,3], Anna Dorfman[2], and Maayan Katzir[3]

[1] Bremen International Graduate School of Social Sciences, Constructor University, Campus Ring 1, Bremen, Germany.

[2] Department of Psychology, Bar-Ilan University, Ramat Gan, Israel.

[3] Conflict Resolution, Management & Negotiation Graduate program, Bar-Ilan University, Ramat Gan, Israel.

Corresponding author: Dora Simunovic (doras@bigsss-bremen.de).

Table of Contents

Supplementary Methods 1 – Translated items. **..3**

Supplementary Methods 2 – Cluster analysis **..7**

Supplementary Methods 3 – Exploratory stepwise linear regression predicting views of the reform. **..8**

Supplementary Methods 4 – Exploratory factor analysis of downstream consequences **.......9**

Supplementary Methods 5 – Deviation from Preregistration and Additional Analyses **.......11**

Supplementary Methods 1 – Translated items.

Original items were presented in Hebrew. We present the English translation of all items and response options (where appropriate) in Table S1. All items related directly to the current reform were composed for the current investigation. Other constructs (trust in institutions, blind vs. constructive patriotism, civic vs. ethnic identity, universalism/benevolence, generalized trust) are credited to their respective authors. Where possible, we used pre-existing, validated Hebrew translations. Otherwise, items were translated to Hebrew by a native Hebrew speaker and back translated to English by a native English speaker. Items marked with an asterisk have been recoded by the authors for ease of analysis and interpretation (e.g., Midgam project documented birth year, which was recoded into age).

Table S1. Translated items.

Construct	Item	Response option/scale	Wave
	**Indices of Polarization**		
Reform-as-threat views	• The reform is a threat to democracy.	1 = Disagree completely<br>7 = Agree completely	T1,T2
Affective polarization[A,F]	*To what extent do you feel the following feelings towards supporters (opponents) of the reform*<br>• Hate<br>• Disgust<br>• Brotherhood (R)<br>• Anger<br>• Fear<br>• Empathy (R)<br>• Affection (R)<br>• Superiority	1 = Not at all<br>7 = To a high extent	T2
Perceived societal Polarization[E]	• To what extent do you think that Israeli society is polarized?	1 = Not at all polarized<br>7 = Extremely polarized	T2
False consensus[F]	*In your opinion, what percentage of Israeli citizens hold each of the following positions*<br>• In favor of the reform<br>• No opinion on the reform<br>• Opposed to the reform	Enter a number between 0 – 100<br>Total percent must sum to 100	T2
Features of democracy[F]	*In your opinion, to what extent each of the following is important for Israel to be a democracy?*<br>• Majority rule<br>• Separation of power between branches of government<br>• Protection of minority rights<br>• Independent and free media<br>• Free elections	1 = Not at all important<br>7 = Important to a high extent	T2

	• Protection of human rights for everyone equally.		
	**Pre-existing socio-psychological characteristics**		
Generalized trust[B]	• Generally speaking, would you say that most people can be trusted, or that you can‘t be too careful in dealing with people?<br>• Do you think that most people would try to take advantage of you if they got the chance, or would they try to be fair?<br>• Would you say that most of the time people try to be helpful or that they are mostly looking out for themselves?	1 = You can’t be too careful<br>4 = It is a mix of both<br>7 = Most people can be trusted<br>1 = Most people would try to take advantage of me<br>4 = It is a mix of both<br>7 = Most people would try to be fair<br>1 = People mostly look out for themselves<br>4 = It is a mix of both<br>7 = People mostly try to be helpful	T2
Universalism / Benevolence[C]	• S/he thinks it is important that every person in the world be treated equally. S/he believes everyone should have equal opportunities in life.<br>• It is important to her/him to listen to people who are different from her/him. Even when s/he disagrees with them, he still wants to understand them.<br>• It’s very important to her/him to help the people around him. S/he wants to care for their well-being.<br>• It’s very important to her/him to be loyal to his friends. S/he wants to devote her/himself to people close to her/him.<br>• S/he strongly believes that people should care for nature. Looking after the environment is important to her/him.	1 = Not like me at all<br>6 = Very much like me	T1
Political Orientation[F]	• What is your political orientation?	1 = Far left<br>5 = Centre<br>9 = Far right	T1,T2
Blind Patriotism[D]	• I would rather be a citizen of Israel than any other country in the world<br>• The world would be a better place if people from other countries were more like Israelis<br>• Generally speaking, Israel is a better country than most other countries<br>• People should support their country even when the country is in the wrong	1 = Disagree completely<br>5 = Agree completely	T1

	• When my country does well in international sports, it makes me proud to be Israeli		
Constructive Patriotism[D]	• There are some things about Israel today that make me feel ashamed of my country<br>• I am often less proud of Israel than I would like to be<br>• The world would be a better place if Israelis acknowledged Israel's shortcomings	1 = Disagree completely<br>5 = Agree completely	T1
Civic identity[D]	• How emotionally close do you feel to Israelis?	1 = Not at all<br>7 = To a very high extent	T1
Ethno-religious identity[D]	• How emotionally close do you feel to Jews?	1 = Not at all<br>7 = To a very high extent	T1
Trust in institutions[D]	*To what extent do you trust the following social institutions?*<br>• Media<br>• Government<br>• Judiciary<br>• Knesset (Parliament)	1 = Distrust completely<br>5 = Trust completely	T1,T2
**Downstream consequences of polarization**			
Conflict management strategies[F]	• I am ready to give up some of my principles so that we do not end up in a civil war over the issue of the reform<br>• I am ready to give up some of my principles related to the issue of the reform for the sake of the unity of the people<br>• It is important to hold dialogue about the reform<br>• It is important to reach a compromise regarding the reform<br>• It is important to reach a broad consensus regarding the reform<br>• When it comes to the reform, there must not be winners and losers<br>• The dialogue will not help to reach an agreement<br>• There is no chance of reaching an agreement regarding the reform	1 = Disagree completely<br>7 = Agree completely	T2
Protest methods[F]	*To what extent is the following acceptable when protesting about the issue of the reform*<br>• General strike<br>• Civil revolt<br>• Disrupting traffic	1 = Not legitimate at all<br>7 = Very legitimate	T2
Protest Control[F]	*To what extent is the following acceptable when dealing with protesters about the issue of the reform*		T2

	• Extreme riot control measures (e.g., water cannons, stun grenades)<br>• Arrests<br>• Police violence against protestors	1 = Not legitimate at all<br>7 = Very legitimate	
Delegitimization of opponents[F]	• They care deeply about democracy (R)<br>• They want to do what they think is best for Israeli society (R)<br>• They are overreacting<br>• They are selfishly using the current situation to push their agenda<br>• They want revenge<br>• They do not understand what democracy is	1 = Not characteristic<br>7 = Highly characteristic	T2
Intergroup violence[F]	• To what extent is violence against protesters from the other side acceptable when protesting about the issue of the reform	1 = Not legitimate at all<br>7 = Very legitimate	T2
Separate[F]	• I support the division of the state.	1 = Disagree completely<br>7 = Agree completely	T2
	**Demographics**		
Gender[F]	• What is your preferred pronoun?	1 = feminine form<br>2 = masculine form	T1,T2
Age[G]*	• Year of Birth		Panel
Education[G]*	• What is your highest level of education?	1 = Highschool<br>2 = Post-high school diploma<br>3 = B.A.<br>4 = M.A.<br>5 = Ph.D.	Panel
Socio-economic status[G]	• Please describe your monthly income.	0 = No income<br>1= Significantly below average<br>2 = Below average<br>3 = Average<br>4 = Above average<br>5 = Significantly above average	Panel
Religiosity[F]	• How religious are you?	1 = Not religious<br>2 = Somewhat religious<br>3 = Religious	T1

NOTE. Sources: **A** = Adapted from: Iyengar, S., Lelkes, Y., Levendusky, M., Malhotra, N. & Westwood, S. J. The Origins and Consequences of Affective Polarization in the United States. *Annu. Rev. Polit. Sci.* **22**, 129–146 (2019). **B** = ESS Round 8: European Social Survey Round 8 Data (2016). *ESS Round 8 Source Questionaire.* London: ESS ERIC Headquarters c/o City University London. doi:10.21338/NSD-ESS8-2016; **C** = Schwartz, S. H. Universalism Values and the Inclusiveness of Our Moral Universe. *Journal of Cross-Cultural Psychology* **38**, 711–728 (2007).; **D** = ISSP Research Group. International Social Survey Programme: National Identity III - ISSP 2013. (2015) doi:10.4232/1.12312; OECD (2017), *OECD Guidelines on Measuring Trust*, OECD Publishing, Paris. https://doi.org/10.1787/9789264278219-en.; **E** = Coppedge, M., Gerring, J., Henrik Knutsen, C., … & Ziblatt, D. 2023. "V-Dem [Country-Year/Country-Date] Dataset v13" Varieties of Democracy (V-Dem) Project. https://doi.org/10.23696/vdemds23. **F** = item composed for the purpose of this investigation. **G** = Midgam Panel item.

Supplementary Methods 2 – Cluster analysis

Figure S1. Dendogram associated with clusters emerging from view of the reform as threat item and political orientation.

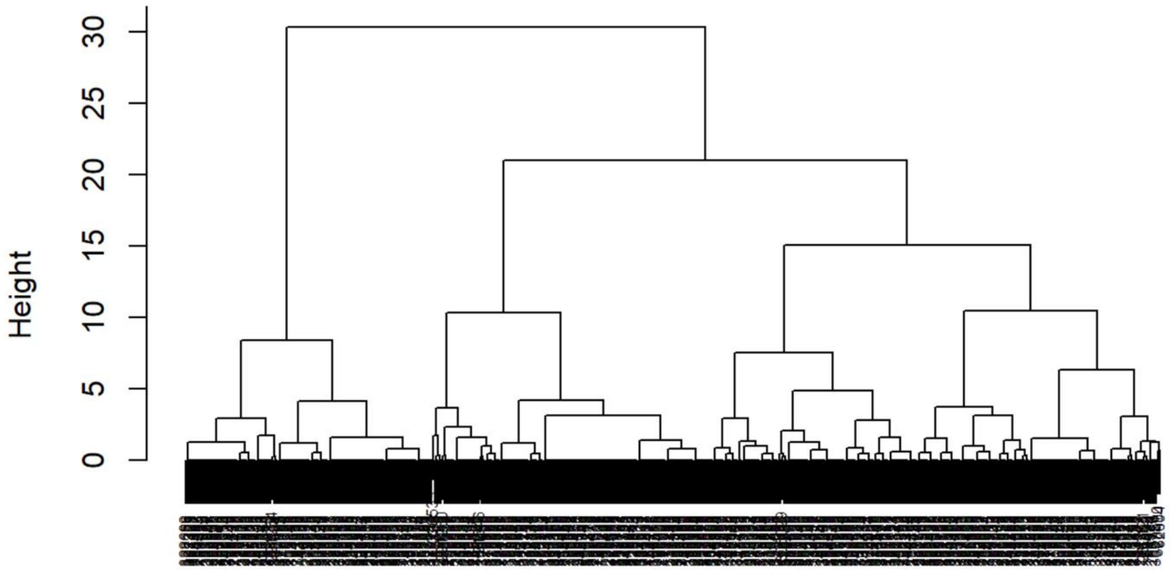

Figure S2. Elbow method of determining optimal number of clusters emerging from view of the reform as threat item and political orientation.

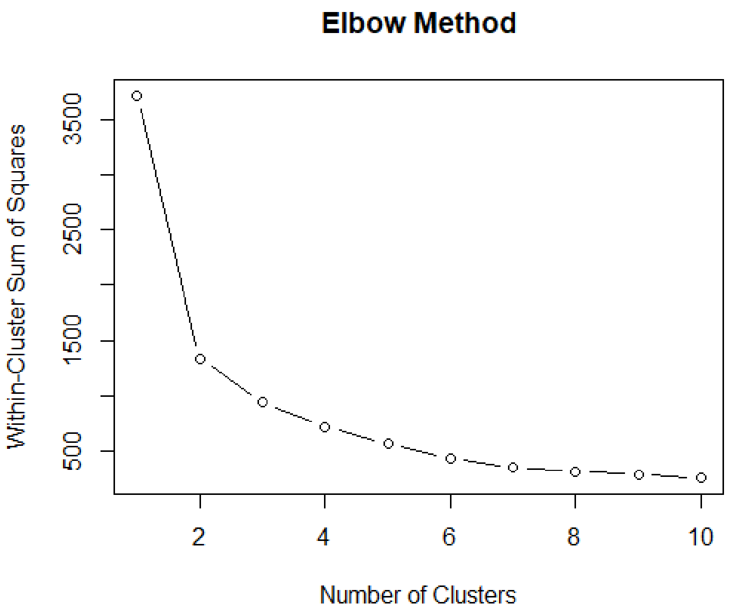

Supplementary Methods 3 – Exploratory stepwise linear regression predicting views of the reform.

Table S2. Predicting views of the reform (stepwise regression)

	Step 1				Step 2				Step 3				Step 4				Step 5			
	B	95%CI	*t*	*p*	*B*	95%CI	*t*	*p*	*B*	95%CI	*t*	*p*	*B*	95%CI	*t*	*p*	*B*	95%CI	*t*	*p*
Intercept	4.32	[4.14, 4.50]	47.91	<.001	4.32	[4.14, 4.50]	48.08	<.001	4.32	[4.16, 4.48]	51.74	<.001	4.32	[4.18, 4.46]	59.49	<.001	4.32	[4.21, 4.43]	77.37	<.001
Gender	0.13	[-0.05, 0.31]	1.46	.144	0.11	[-0.07, 0.29]	1.23	.219	0.12	[-0.04, 0.29]	1.46	.145	0.13	[-0.01, 0.28]	1.78	.075	**0.12**	**[0.01, 0.24]**	**2.15**	**.032**
Age	0.03	[-0.15, 0.22]	0.35	.724	0.02	[-0.16, 0.21]	0.26	.798	0.05	[-0.13, 0.22]	0.54	.587	0.12	[-0.03, 0.28]	1.59	.112	-0.01	[-0.13, 0.11]	-0.10	.917
Education	-0.02	[-0.20, 0.17]	-0.18	.859	-0.03	[-0.21, 0.16]	-0.29	.772	-0.08	[-0.26, 0.09]	-0.96	.340	-0.09	[-0.24, 0.06]	-1.14	.256	**-0.12**	**[-0.23, 0.00]**	**-1.96**	**.050**
SES	0.17	[-0.02, 0.36]	1.76	.079	0.17	[-0.03, 0.36]	1.69	.092	0.16	[-0.02, 0.33]	1.71	.087	**0.16**	**[0.01, 0.32]**	**2.05**	**.041**	0.08	[-0.04, 0.20]	1.31	.191
Religiosity	**-1.08**	**[-1.26, -0.90]**	**-11.82**	**<.001**	**-1.09**	**[-1.27, -0.90]**	**-11.65**	**<.001**	**-0.87**	**[-1.05, -0.69]**	**-9.57**	**<.001**	**-0.54**	**[-0.70, -0.37]**	**-6.34**	**<.001**	**-0.38**	**[-0.50, -0.25]**	**-5.70**	**<.001**
Wave: T1 vs. T2	**-0.11**	**[-0.18, -0.05]**	**-3.40**	**.001**	**-0.11**	**[-0.18, -0.05]**	**-3.40**	**.001**	**-0.12**	**[-0.18, -0.05]**	**-3.46**	**.001**	**-0.12**	**[-0.18, -0.05]**	**-3.46**	**.001**	**-0.18**	**[-0.25, -0.11]**	**-5.26**	**<.001**
Universalism/Benevolence					0.17	[-0.01, 0.34]	1.82	.069	0.07	[-0.10, 0.23]	0.76	.446	0.04	[-0.11, 0.18]	0.47	.640	-0.02	[-0.13, 0.10]	-0.27	.788
General trust					0.04	[-0.15, 0.22]	0.41	.681	-0.00	[-0.17, 0.17]	-0.02	.985	0.06	[-0.09, 0.22]	0.82	.413	-0.05	[-0.17, 0.06]	-0.90	.366
Political affiliation									**-0.58**	**[-0.72, -0.43]**	**-7.75**	**<.001**	**-0.36**	**[-0.50, -0.22]**	**-5.04**	**<.001**	**-0.17**	**[-0.30, -0.05]**	**-2.79**	**.005**
Civic identity													**0.46**	**[0.27, 0.65]**	**4.80**	**<.001**	**0.25**	**[0.10, 0.40]**	**3.27**	**.001**
Ethno-religious identity													**-0.34**	**[-0.54, -0.13]**	**-3.23**	**.001**	-0.14	[-0.30, 0.02]	-1.72	.086
Constructive patriotism													**0.80**	**[0.64, 0.97]**	**9.43**	**<.001**	**0.45**	**[0.32, 0.59]**	**6.59**	**<.001**
Blind patriotism													**-0.37**	**[-0.57, -0.17]**	**-3.66**	**<.001**	**-0.20**	**[-0.35, -0.04]**	**-2.50**	**.013**
Trust in judiciary																	**0.57**	**[0.45, 0.70]**	**8.95**	**<.001**
Trust in government																	**-0.62**	**[-0.75, -0.49]**	**-9.13**	**<.001**
Trust in parliament																	0.01	[0.30, 0.53]	0.12	.903
Trust in media																	**0.42**	**[-0.11, 0.13]**	**6.99**	**<.001**
AIC	3872.29				3878.58				3830.94				3703.99				3486.45			
BIC	3916.28				3932.35				3889.593				3782.19				3587.32			
Adjusted R^2	.22				.23				.29				.46				.63			
Sigma	1.03				1.03				1.05				1.04				1.05			

Note. Significant effects are **bolded**. Eight participants who had missing data in the generalized trust items were excluded from this analysis.

Brief reasoning for ordering of exploratory models: The above stepwise linear regression was an exploratory tool trying to understand the contributions of various factors on views of the reform. Demographics were entered first as common control variables. The model proceeded by adding within-person characteristics, and then group-level attitudes, including trust in institutions in the final step. We acknowledge that this was not the only possible, logical way to order the variables.

Supplementary Methods 4 – Exploratory factor analysis of downstream consequences

We ran an exploratory factor analysis which included all downstream consequences items. The resulting scree-plot indicated three factors (see Figure S3).

One factor included *conflict management strategies* items.

The second factor included all of the items related to *delegitimization of political opponents* (e.g., "They do not understand democracy").

The third factor included only the first three items related to *protest methods*. One item related to protest control (arrests of protesters) also loaded on this factor, with negative loading. However, because this protest control item did not conceptually align with endorsement of extreme protest methods, we did not include it in the protest methods index.

The arrests of protesters item was conceptually related to the other *protest control* items (use of stun grenades and watercannons, police violence against protesters). These three protest control items showed high levels of inter-item reliability ($\alpha_{Cronbach}$ = .81). Therefore, we decided to combine into a fourth index – "protest control".

Three items ("There is no chance to reach an agreement about the reform," "I support the division of the state," and "To what extent is violence against protesters from the other side acceptable when protesting about the issue of the reform") did not load on and were not strongly conceptually related to any of four factors. Therefore, we removed them from all further analyses.

Figure S3. EFA Downstream consequences Scree plot.

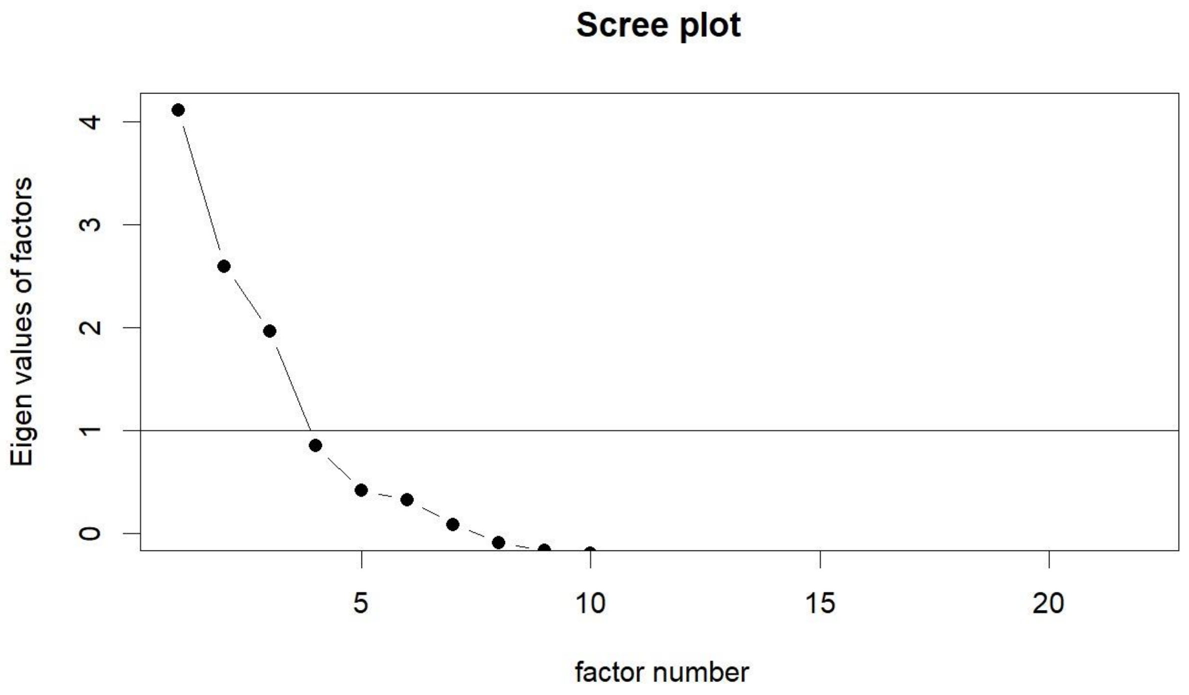

Table S3. Exploratory factor analysis and scree plot: Downstream consequences of polarization.

	Factor 1 Conflict management	Factor 2 Delegitimization	Factor 3 Protest methods
I am ready to give up some of my principles so that we do not end up in a civil war over the issue of the reform	.54		
I am ready to give up some of my principles related to the issue of the reform for the sake of the unity of the people	.42		
It is important to hold dialogue about the reform	.85		
It is important to reach a compromise regarding the reform	.82		
It is important to reach a broad consensus regarding the reform	.85		
When it comes to the reform, there must not be winners and losers	.69		
The dialogue will not help to reach an agreement *(reversed)*	.42		
There is no chance of reaching an agreement regarding the reform *(reversed)*			
They care deeply about democracy *(reversed)*		.60	
They want to do what they think is best for Israeli society *(reversed)*		.41	
They are overreacting		.81	
They are selfishly using the current situation to push their agenda		.81	
They want revenge		.77	
They do not understand what democracy is		.71	
General strike			.87
Civil revolt			.84
Disrupting traffic			.84
To what extent is violence against protesters from the other side acceptable when protesting about the issue of the reform			
Extreme riot control measures (e.g., water cannons, stun grenades)			
Arrests			-.41
Police violence against protestors			

Supplementary Methods 5 – Deviation from Preregistration and Additional Analyses

In addition to the pre-registered exclusion we applied, we also pre-registered (https://aspredicted.org/JMP_QKT) exclusions based on identical responses to opposing items. Upon additional review we realized that similar or even identical responses to these items do not necessarily indicate low-quality responses that merit exclusion (e.g., a person who disagrees with both the item *The reform is crucial for Israel's democracy* and the item *The reform is a threat to democracy*), as these items are not logically opposing. Moreover, excluding participants based on this criterion reduced the sample by 40.6%. Therefore, we decided to forgo this exclusion criterion. For transparency, we reproduced the core analyses using the original pre-registered criteria and present the key tables (Tables S4 - S7) and figures (Figures S4 - S7) below. To replicate the analyses reported here, please use R code "prereg_exclude_LMM_OSF_R1_final" and "prereg_exclude_Downstream_OSF_R1_final" with datafile "data_for_preregexclusion" from: https://osf.io/uscyr/?view_only=967e56221a9a47568b4841b1a6e3f421

Furthermore, we preregistered the two items mentioned in the example above to assess view of the reform as a threat to democracy. Upon consideration, we realized that only the item *The reform is a threat to democracy* directly assesses view of the reform as a threat, and therefore decided to drop the item *The reform is crucial for Israel's democracy*.

Our preregistration document also included items unrelated to the current investigation (e.g., wise reasoning, impact of protests on Israel's economy).

Table S4. Predicting views of the reform as threat.

Variable	*B*	95%CI	*t*	*p*
Intercept	4.56	[4.42, 4.71]	62.56	<.001
Gender	0.13	[-0.02, 0.28]	1.65	.100
Age	-0.05	[-0.20, 0.11]	-0.61	.542
Education	-0.15	[-0.30, 0.01]	-1.90	.058
SES	0.03	[-0.13, 0.18]	0.34	.736
Religiosity	**-0.35**	**[-0.52, -0.18]**	**-4.10**	**<.001**
Wave (T1 vs. T2)	**-0.19**	**[-0.28, -0.11]**	**-4.44**	**<.001**
Universalism/Benevolence	-0.07	[-0.23, 0.08]	-0.96	.339
Generalized trust	-0.09	[-0.24, 0.06]	-1.20	.230
Political affiliation	**-0.24**	**[-0.40, -0.08]**	**-3.00**	**.003**
Civic identity	**0.33**	**[0.14, 0.51]**	**3.43**	**.001**
Ethno-religious identity	-0.04	[-0.25, 0.17]	-0.39	.694
Constructive patriotism	**0.42**	**[0.25, 0.59]**	**4.86**	**<.001**
Blind patriotism	-0.19	[-0.39, 0.02]	-1.80	.072
Trust in judiciary	**0.58**	**[0.43, 0.74]**	**7.50**	**<.001**
Trust in government	**-0.65**	**[-0.83, -0.48]**	**-7.54**	**<.001**
Trust in parliament	**0.34**	**[0.20, 0.48]**	**4.68**	**<.001**
Trust in media	0.03	[-0.12, 0.18]	0.42	.674
Interaction with Wave (T1 v. T2)				
*Trust in judiciary	0.08	[-0.03, 0.19]	1.42	.156
*Trust in government	-0.04	[-0.16, 0.09]	-0.58	.561
*Trust in parliament	-0.08	[-0.21, 0.05]	-1.25	.212
*Trust in media	0.06	[-0.05, 0.17]	1.02	.308
AIC		2163.08		
BIC		2268.37		
Adjusted R^2		.59		
Sigma		1.02		

Note. Significant effects are **bolded**. Both exclusion criteria applied (*N* = 297).

Table S5. Predicting issue-based, affective, and perceived polarization.

	Model 1 Issue-Based Polarization				Model 2 Affective Polarization				Model 3 Perceived Societal Polarization			
Main effects	*B*	95%CI	*t*	*p*	*B*	95%CI	*t*	*p*	*B*	95%CI	*t*	*p*
Intercept	2.22	[1.80,2.65]	10.20	<.001	4.20	[3.60,4.81]	13.66	<.001	5.82	[5.23,6.40]	19.49	<.001
Gender	-0.08	[-0.19,0.03]	-1.50	.134	0.07	[-0.08,0.22]	0.93	.351	0.03	[-0.12,0.17]	0.37	.711
Age	0.06	[-0.05,0.17]	1.02	.308	-0.04	[-0.20,0.11]	-0.53	.597	0.11	[-0.04,0.26]	1.47	.142
Education	0.01	[-0.10,0.12]	0.13	.900	0.06	[-0.09,0.21]	0.75	.453	0.11	[-0.03,0.26]	1.52	.130
SES	0.03	[-0.08,0.15]	0.57	.567	-0.01	[-0.16,0.15]	-0.09	.930	0.00	[-0.15,0.15]	0.03	.974
Religiosity	-0.08	[-0.20,0.04]	-1.26	.208	-0.07	[-0.24,0.10]	-0.84	.399	-0.13	[-0.30,0.03]	-1.57	.119
Universalism/Benevolence	0.03	[-0.08,0.13]	0.48	.628	**-0.16**	**[-0.30,-0.01]**	**-2.07**	**.039**	**0.21**	**[0.07,0.36]**	**2.93**	**.004**
Generalized trust	-0.01	[-0.12,0.10]	-0.12	.902	-0.13	[-0.28,0.02]	-1.73	.084	-0.01	[-0.16,0.14]	-0.13	.894
Civic identity	0.10	[-0.04,0.23]	1.40	.161	0.01	[-0.17,0.20]	0.13	.896	-0.03	[-0.21,0.15]	-0.37	.714
Ethno-religious identity	0.01	[-0.14,0.16]	0.15	.881	-0.10	[-0.31,0.10]	-1.02	.311	-0.09	[-0.28,0.11]	-0.85	.394
Constructive patriotism	0.10	[-0.03,0.22]	1.50	.134	0.14	[-0.03,0.31]	1.65	.099	0.12	[-0.05,0.29]	1.42	.157
Blind patriotism	0.04	[-0.10,0.19]	0.56	.578	0.01	[-0.19,0.21]	0.10	.922	-0.04	[-0.23,0.15]	-0.42	.672
Trust in judiciary	-0.11	[-0.22,0.00]	-1.89	.059	-0.12	[-0.31,0.07]	-1.22	.223	-0.03	[-0.22,0.15]	-0.34	.730
Trust in government	-0.02	[-0.15,0.10]	-0.38	.703	0.14	[-0.07,0.35]	1.34	.182	-0.10	[-0.30,0.10]	-0.96	.338
Trust in parliament	-0.01	[-0.12,0.09]	-0.24	.809	-0.07	[-0.26,0.13]	-0.68	.499	-0.04	[-0.23,0.14]	-0.48	.633
Trust in media	0.05	[-0.05,0.16]	1.04	.300	-0.08	[-0.26,0.10]	-0.91	.365	0.00	[-0.18,0.17]	-0.05	.960
Cluster	-0.08	[-0.37,0.21]	-0.56	.575	-0.01	[-0.42,0.41]	-0.04	.969	-0.16	[-0.56,0.24]	-0.80	.423
Wave (T1 v. T2)	0.00	[-0.06,0.06]	-0.08	.932								
Interactions with Cluster												
*Universalism/Ben	-0.05	[-0.24,0.13]	-0.58	.560	0.02	[-0.26,0.30]	0.16	.880	-0.07	[-0.35,0.22]	-0.45	.650
Anti-Reform	-0.01	[-0.14,0.12]	-0.19	.851	-0.23	[-0.42,-0.03]	-2.31	.022	0.20	[0.00,0.40]	1.96	.051
Pro-Reform	-0.07	[-0.20,0.07]	-0.98	.328	-0.20	[-0.40,0.00]	-2.00	.047	0.13	[-0.08,0.34]	1.25	.211
*Generalized trust	0.01	[-0.18,0.20]	0.12	.903	-0.16	[-0.44,0.13]	-1.09	.275	-0.10	[-0.39,0.19]	-0.69	.492
Anti-Reform	-0.01	[-0.14,0.11]	-0.18	.857	-0.08	[-0.26,0.11]	-0.80	.426	0.03	[-0.17,0.22]	0.27	.784
Pro-Reform	0.00	[-0.14,0.14]	0.00	.998	-0.23	[-0.44,-0.02]	-2.18	.030	-0.07	[-0.29,0.14]	-0.68	.498
*Civic identity	**-0.43**	**[-0.66,-0.19]**	**-3.54**	**<.001**	-0.13	[-0.49,0.22]	-0.73	.468	0.13	[-0.24,0.49]	0.68	.500
Anti-Reform	**0.27**	**[0.12,0.41]**	**3.59**	**<.001**	0.07	[-0.15,0.29]	0.63	.530	-0.10	[-0.32,0.13]	-0.83	.406

Pro-Reform	-0.16	[-0.35,0.03]	-1.68	.094	-0.06	[-0.34,0.22]	-0.43	.669	0.03	[-0.26,0.32]	0.21	.837
*Ethno-religious identity	0.20	[-0.10,0.50]	1.32	.187	0.04	[-0.41,0.48]	0.16	.875	0.17	[-0.30,0.63]	0.71	.479
Anti-Reform	0.04	[-0.11,0.18]	0.50	.617	-0.02	[-0.23,0.20]	-0.15	.884	-0.05	[-0.27,0.18]	-0.42	.676
Pro-Reform	0.24	[-0.03,0.50]	1.77	.078	0.02	[-0.38,0.41]	0.10	.923	0.12	[-0.29,0.53]	0.57	.569
*Constructive patriotism	**-0.37**	**[-0.58,-0.16]**	**-3.43**	**.001**	**-0.44**	**[-0.76,-0.12]**	**-2.69**	**.008**	-0.15	[-0.48,0.18]	-0.88	.381
Anti-Reform	**0.25**	**[0.11,0.40]**	**3.39**	**.001**	**0.33**	**[0.12,0.55]**	**3.01**	**.003**	0.18	[-0.04,0.41]	1.60	.112
Pro-Reform	-0.12	[-0.27,0.04]	-1.51	.132	-0.10	[-0.34,0.13]	-0.87	.383	0.04	[-0.20,0.28]	0.29	.771
*Blind patriotism	**0.27**	**[0.00,0.53]**	**1.98**	**.048**	0.13	[-0.27,0.52]	0.63	.530	0.21	[-0.20,0.61]	1.01	.315
Anti-Reform	-0.12	[-0.28,0.05]	-1.42	.157	-0.10	[-0.34,0.15]	-0.78	.438	-0.15	[-0.40,0.10]	-1.18	.238
Pro-Reform	0.15	[-0.06,0.35]	1.41	.160	0.03	[-0.28,0.34]	0.19	.852	0.06	[-0.26,0.37]	0.34	.731
*Trust in judiciary	**-0.61**	**[-0.82,-0.40]**	**-5.69**	**<.001**	**-0.56**	**[-0.93,-0.2]**	**-3.02**	**.003**	**-0.44**	**[-0.82,-0.06]**	**-2.27**	**.024**
Anti-Reform	**0.17**	**[0.04,0.31]**	**2.50**	**.013**	0.18	[-0.05,0.41]	1.54	.125	0.16	[-0.08,0.40]	1.29	.200
Pro-Reform	**-0.43**	**[-0.59,-0.28]**	**-5.34**	**<.001**	**-0.38**	**[-0.67,-0.1]**	**-2.64**	**.009**	-0.28	[-0.57,0.01]	-1.87	.062
*Trust in government	**0.52**	**[0.28,0.76]**	**4.30**	**<.001**	**0.66**	**[0.26,1.07]**	**3.24**	**.001**	0.12	[-0.30,0.53]	0.54	.587
Anti-Reform	**-0.34**	**[-0.5,-0.17]**	**-3.95**	**<.001**	**-0.30**	**[-0.58,-0.03]**	**-2.17**	**.031**	-0.24	[-0.53,0.05]	-1.65	.099
Pro-Reform	**0.18**	**[0.02,0.35]**	**2.16**	**.031**	**0.36**	**[0.07,0.65]**	**2.44**	**.015**	-0.12	[-0.43,0.18]	-0.81	.417
*Trust in parliament	0.00	[-0.20,0.21]	0.01	.989	0.18	[-0.19,0.54]	0.97	.335	0.25	[-0.13,0.62]	1.29	.197
Anti-Reform	-0.01	[-0.14,0.12]	-0.16	.870	-0.11	[-0.33,0.12]	-0.94	.348	-0.11	[-0.34,0.12]	-0.96	.337
Pro-Reform	-0.01	[-0.16,0.14]	-0.12	.902	0.07	[-0.22,0.36]	0.49	.624	0.13	[-0.16,0.43]	0.89	.375
*Trust in media	-0.13	[-0.33,0.06]	-1.35	.176	-0.32	[-0.67,0.03]	-1.81	.072	-0.08	[-0.44,0.29]	-0.42	.676
Anti-Reform	0.14	[0.02,0.26]	2.21	.027	0.06	[-0.15,0.26]	0.56	.579	0.05	[-0.16,0.26]	0.44	.664
Pro-Reform	0.00	[-0.15,0.15]	0.03	.978	-0.26	[-0.55,0.02]	-1.82	.070	-0.03	[-0.33,0.26]	-0.20	.840
*T1 vs. T2	0.06	[-0.06,0.18]	0.93	.355								
Anti-Reform	-0.01	[-0.14,0.12]	-0.16	.870								
Pro-Reform	-0.01	[-0.16,0.14]	-0.12	.902								
AIC		1712.41				944.39				964.85		
BIC		1848.41				1047.81				1068.27		
Adjusted R^2		.24				.18				.15		
Sigma		0.73				1.13				1.17		

Note. Significant effects are **bolded**. For variables that significantly interacted with cluster, the interaction term and simple slopes are bolded, but corresponding significant main effects are not. Both exclusion criteria applied (N = 297).

Table S6. Predicting downstream consequences of polarization: Protest methods, protest control, conflict management strategies, and delegitimization of political opponents

	Protest Methods				Protest Control				Conflict Management				Delegitimization			
Main effects	*B*	95%CI	*t*	*p*	*B*	95%CI	*t*	*p*	*B*	95%CI	*t*	*p*	*B*	95%CI	*t*	*p*
Intercept	4.14	[3.88,4.40]	31.05	<.001	2.51	[2.28,2.74]	21.48	<.001	4.96	[4.76,5.15]	50.42	<.001	4.81	[4.63,4.98]	55.10	<.001
Gender	**-0.14**	**[-0.33,0.05]**	**-1.45**	**.149**	**-0.24**	**[-0.41,-0.07]**	**-2.84**	**.005**	0.07	[-0.07,0.21]	0.97	.332	-0.08	[-0.2,0.05]	-1.21	.228
Age	-0.10	[-0.29,0.10]	-0.94	.346	-0.17	[-0.34,0.01]	-1.89	.060	0.09	[-0.06,0.24]	1.21	.228	**0.14**	**[0.01,0.27]**	**2.14**	**.033**
Education	0.10	[-0.10,0.29]	0.97	.335	-0.09	[-0.27,0.08]	-1.05	.292	0.08	[-0.07,0.22]	1.04	.297	-0.08	[-0.21,0.05]	-1.26	.209
SES	0.00	[-0.20,0.20]	-0.01	.996	0.16	[-0.02,0.33]	1.77	.078	-0.05	[-0.20,0.09]	-0.72	.474	0.05	[-0.08,0.18]	0.70	.484
Religiosity	-0.17	[-0.38,0.05]	-1.52	.131	0.15	[-0.04,0.34]	1.55	.123	0.03	[-0.13,0.19]	0.34	.734	0.12	[-0.02,0.26]	1.69	.092
Issue-based pol.	0.17	[-0.04,0.37]	1.59	.114	-0.09	[-0.27,0.09]	-0.94	.347	-0.11	[-0.27,0.04]	-1.48	.140	**0.30**	**[0.16,0.43]**	**4.34**	**<.001**
Affective pol.	0.14	[-0.06,0.34]	1.36	.175	0.18	[0.01,0.36]	2.05	.041	**-0.26**	**[-0.40,-0.11]**	**-3.39**	**.001**	**0.70**	**[0.57,0.83]**	**10.55**	**<.001**
Perceived pol.	**0.20**	**[0.00,0.40]**	**1.98**	**.049**	0.00	[-0.17,0.18]	0.05	.961	0.01	[-0.13,0.16]	0.19	.850	**0.20**	**[0.06,0.33]**	**2.93**	**.004**
Cluster	**-1.84**	**[-2.27,-1.41]**	**-8.37**	**<.001**	**1.16**	**[0.78,1.54]**	**6.01**	**<.001**	**-0.35**	**[-0.67,-0.03]**	**-2.16**	**.032**	0.23	[-0.06,0.51]	1.57	.117
Interactions with cluster																
*Issue-based pol.	**-1.14**	**[-1.51,-0.78]**	**-6.11**	**<.001**	**0.69**	**[0.35,1.03]**	**3.99**	**<.001**	-0.06	[-0.36,0.25]	-0.38	.706	0.22	[-0.04,0.49]	1.65	.101
Anti-Reform	**0.66**	**[0.41,0.91]**	**5.19**	**<.001**	**-0.38**	**[-0.61,-0.15]**	**-3.24**	**.001**	-0.09	[-0.30,0.11]	-0.87	.383	0.20	[0.02,0.38]	2.16	.032
Pro-Reform	**-0.49**	**[-0.76,-0.21]**	**-3.49**	**.001**	**0.31**	**[0.06,0.56]**	**2.41**	**.017**	-0.15	[-0.38,0.08]	-1.30	.193	0.42	[0.22,0.61]	4.19	<.001
*Affective pol.	**-0.50**	**[-0.86,-0.14]**	**-2.75**	**.006**	**0.43**	**[0.10,0.77]**	**2.57**	**.011**	-0.01	[-0.31,0.28]	-0.08	.937	0.11	[-0.15,0.37]	0.87	.388
Anti-Reform	**0.34**	**[0.10,0.59]**	**2.75**	**.006**	0.00	[-0.22,0.23]	0.03	.979	-0.25	[-0.45,-0.05]	-2.47	.014	0.65	[0.48,0.83]	7.34	<.001
Pro-Reform	-0.16	[-0.43,0.10]	-1.20	.232	**0.44**	**[0.19,0.68]**	**3.50**	**.001**	-0.26	[-0.48,-0.05]	-2.38	.018	0.77	[0.57,0.96]	7.81	<.001
*Perceived pol.	-0.33	[-0.69,0.03]	-1.79	.075	0.18	[-0.15,0.52]	1.09	.279	-0.16	[-0.45,0.14]	-1.03	.303	**-0.26**	**[-0.52,0.00]**	**-1.99**	**.047**
Anti-Reform	0.35	[0.10,0.59]	2.78	.006	-0.08	[-0.30,0.15]	-0.66	.513	0.09	[-0.12,0.29]	0.83	.406	**0.32**	**[0.14,0.50]**	**3.54**	**<.001**
Pro-Reform	0.02	[-0.24,0.28]	0.15	.882	0.11	[-0.14,0.35]	0.87	.388	-0.07	[-0.29,0.15]	-0.63	.529	0.06	[-0.14,0.25]	0.57	.568
AIC		1096.49				1049.44				981.75				885.51		
BIC		1148.34				1101.29				1033.60				937.18		
Adjusted R^2		.45				.29				.06				.42		
Sigma		1.47				1.36				1.21				1.05		
N		300				300				300				296		

Note. Both exclusion criteria applied.

Figure S4: Distributions of responses to the statement "Reform is a threat to democracy" at T1 and T2.

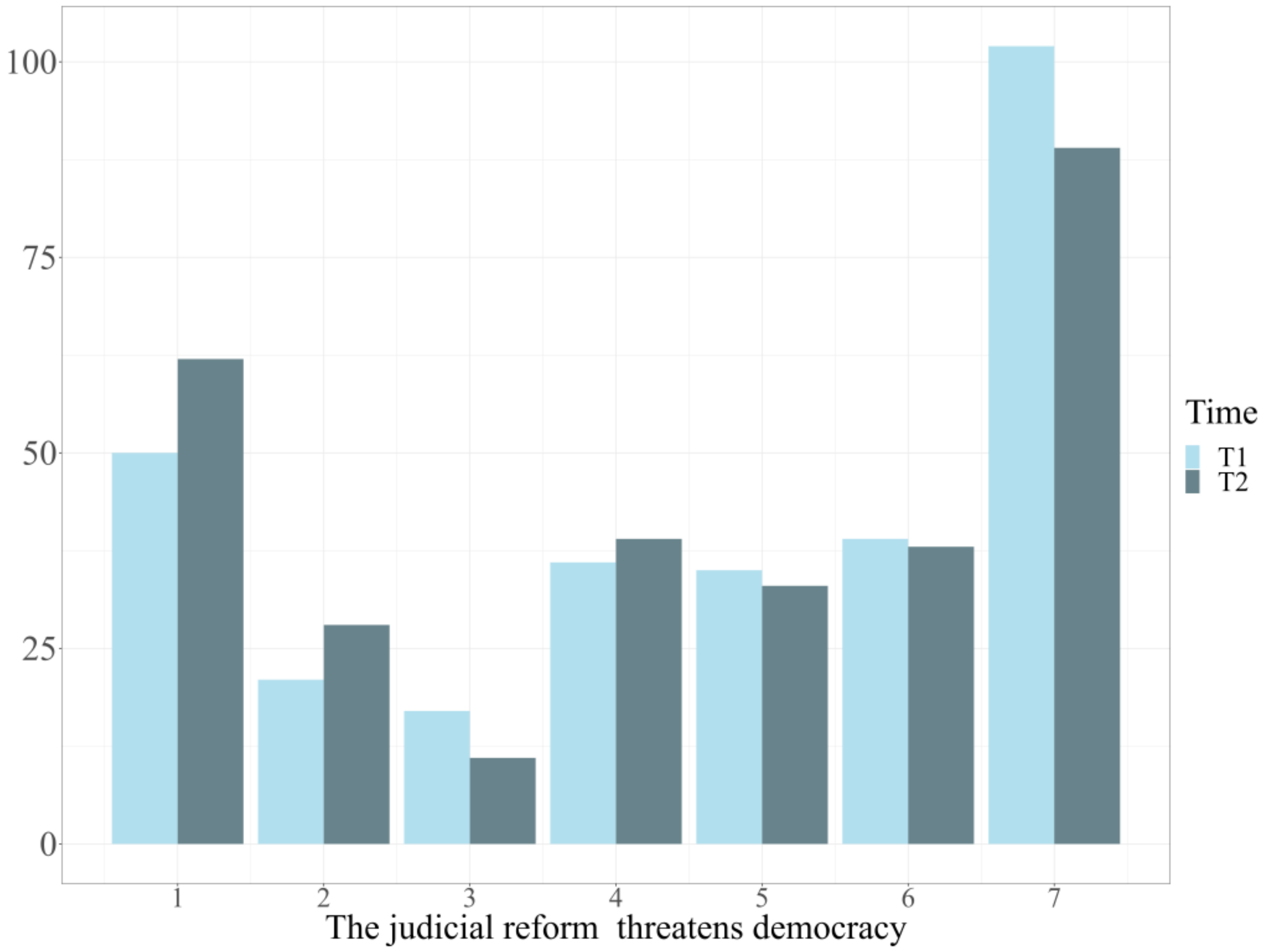

Time 1 (T1) distribution appears in light blue, Time 2 (T2) distribution appears in dark blue. Responses ranged from 1 = *Disagree completely* to 7 = *Agree completely.* Both exclusion criteria applied (*N* = 300).

Table S7. Comparison of pro- and anti-reform clusters on main study variables.

Variable	*t*	df	95%CI	*p*	Cohen's *d*	*M*(Anti-reform)	*M*(Pro-reform)
Universalism/Benevolence	1.32	255.92	[-0.08, 0.39]	.187	0.16	4.59	4.44
Generalized.trust	-0.94	254.86	[-0.48, 0.17]	.348	0.11	3.98	4.13
Civic identity	-1.54	282.11	[-0.64, 0.08]	.124	0.18	5.27	5.55
Ethno-religious identity	**-6.90**	**294.51**	**[-1.37, -0.76]**	**<.001**	**0.76**	**5.26**	**6.32**
Constructive patriotism	**7.87**	**258.94**	**[0.56, 0.94]**	**<.001**	**0.93**	**3.86**	**3.11**
Blind patriotism	**-6.95**	**277.37**	**[-0.77, -0.43]**	**<.001**	**0.81**	**3.23**	**3.83**
Trust in Judiciary	**9.55**	**269.61**	**[0.87, 1.33]**	**<.001**	**1.12**	**3.24**	**2.14**
Trust in Government	**-12.16**	**218.56**	**[-1.43, -1.03]**	**<.001**	**1.49**	**1.62**	**2.85**
Trust in Parliament	**-5.09**	**260.65**	**[-0.68, -0.3]**	**<.001**	**0.60**	**1.92**	**2.41**
Trust in Media	**7.44**	**290.67**	**[0.54, 0.93]**	**<.001**	**0.85**	**2.69**	**1.95**
Issue-based polarization	**2.25**	**272.07**	**[0.03, 0.51]**	**.026**	**0.26**	**2.09**	**1.82**
Affective polarization	-0.11	267.90	[-0.3, 0.27]	.911	0.01	4.18	4.20
Perceived polarization	**4.32**	**266.07**	**[0.35, 0.93]**	**<.001**	**0.51**	**5.83**	**5.20**
Conflict management strategies	**2.12**	**220.77**	**[0.02, 0.62]**	**.035**	**0.26**	**4.95**	**4.62**
Protest methods	**11.20**	**294.66**	**[1.76, 2.51]**	**<.001**	**1.27**	**4.26**	**2.13**
Protest control	**-7.48**	**229.3**	**[-1.68, -0.98]**	**<.001**	**0.91**	**2.43**	**3.77**
Delegitimization	-1.02	262.73	[-0.49, 0.16]	.310	0.12	4.83	5.00

Note. Both exclusion criteria applied ($N = 300$).

Figure S5: Distribution of political orientation by cluster.

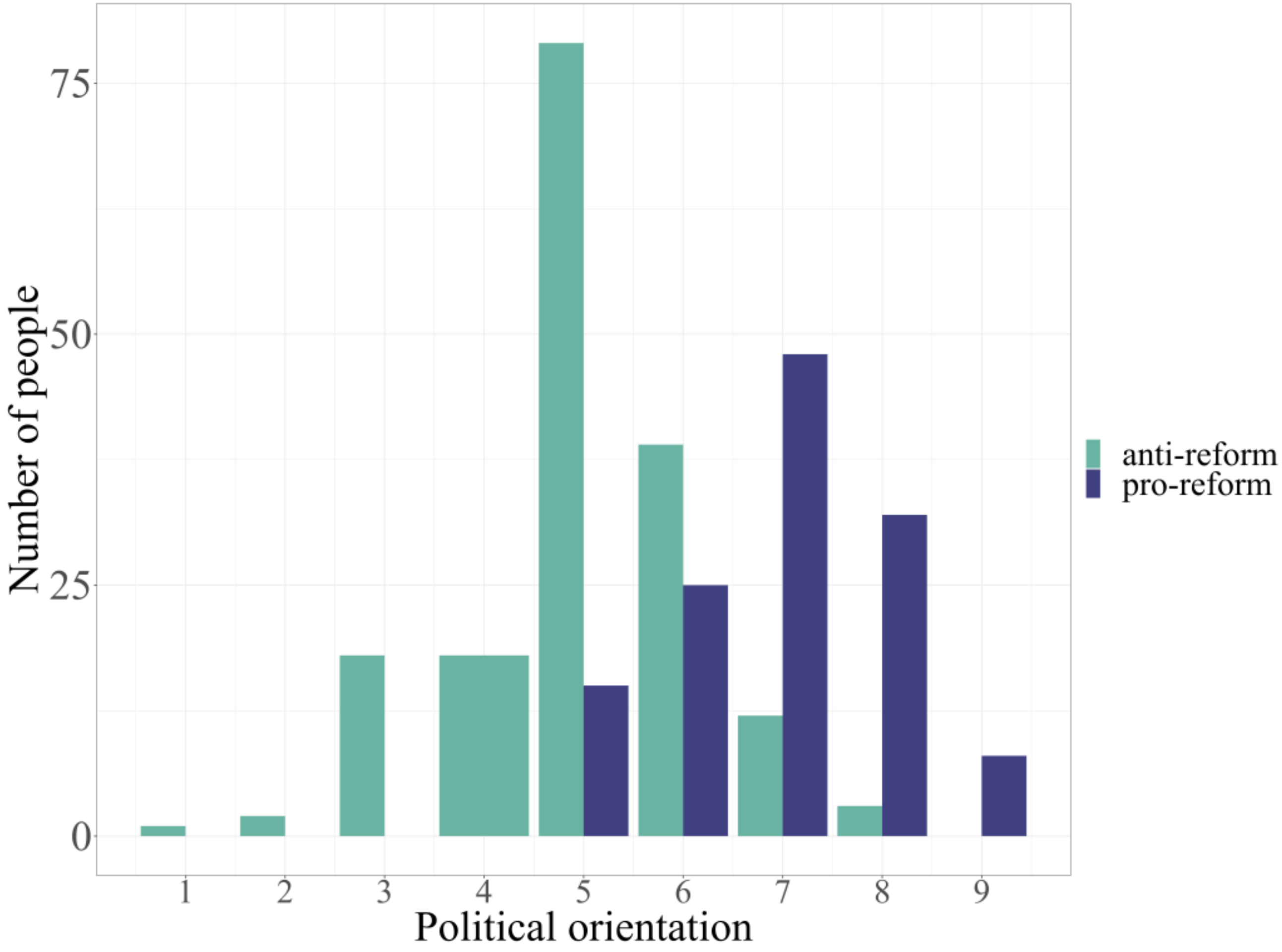

Participants (*N* = 300) indicated their political orientation on a 9-point scale (1= *extreme left*, 5 = *center*, 9 = *extreme right*). Pro-reform cluster appears in purple, anti-reform cluster appears in green. Both exclusion criteria applied.

Figure S6: False consensus by cluster and issue-based polarization.

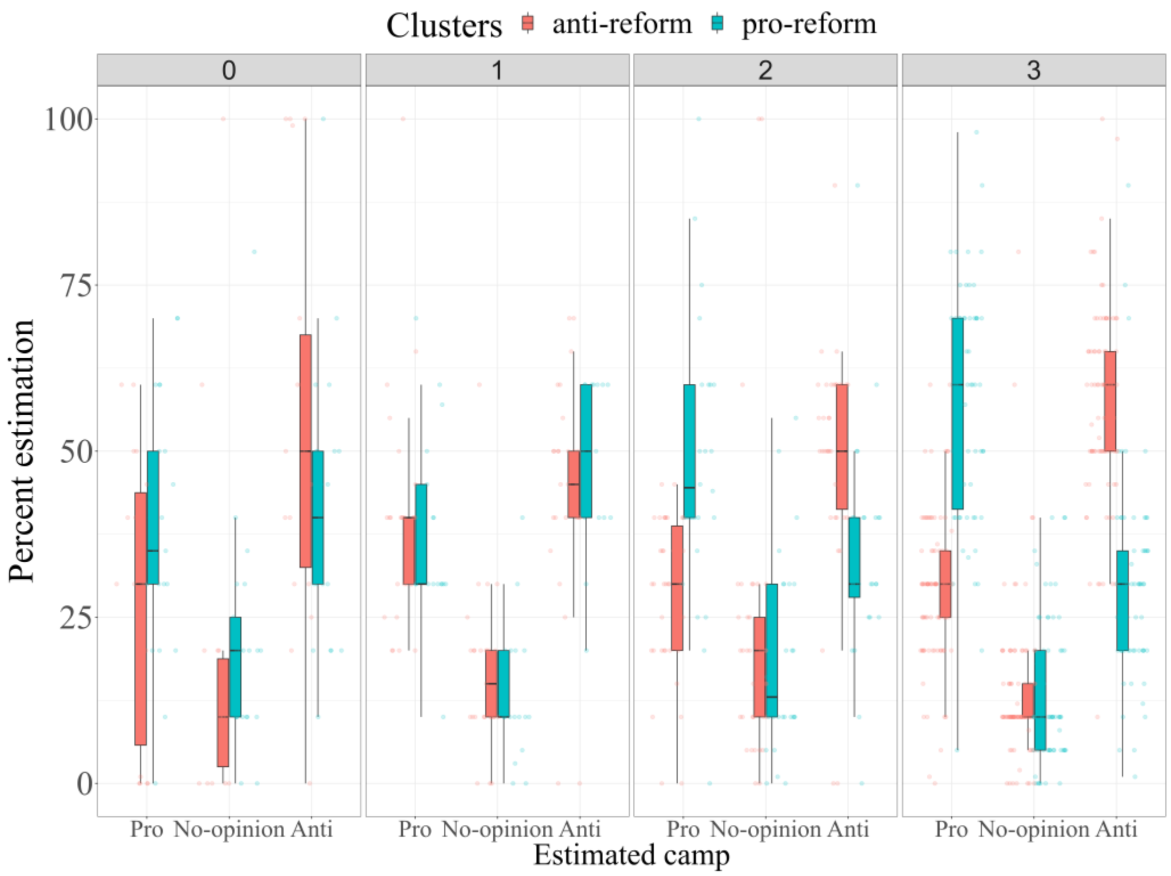

Percent estimation of camp as a function of estimated camp (Pro = pro-reform, No-opinion, Anti = anti-reform), cluster (red: pro-reform, green: anti-reform) and level of issue-based polarization (0 = *neutral view*, 1 = *moderate view*, 2 = *extreme view*, 3 = *most extreme view*). Error bars denote 95% CIs. Both exclusion criteria applied (N = 300).

Figure S7: Importance of democracy features.

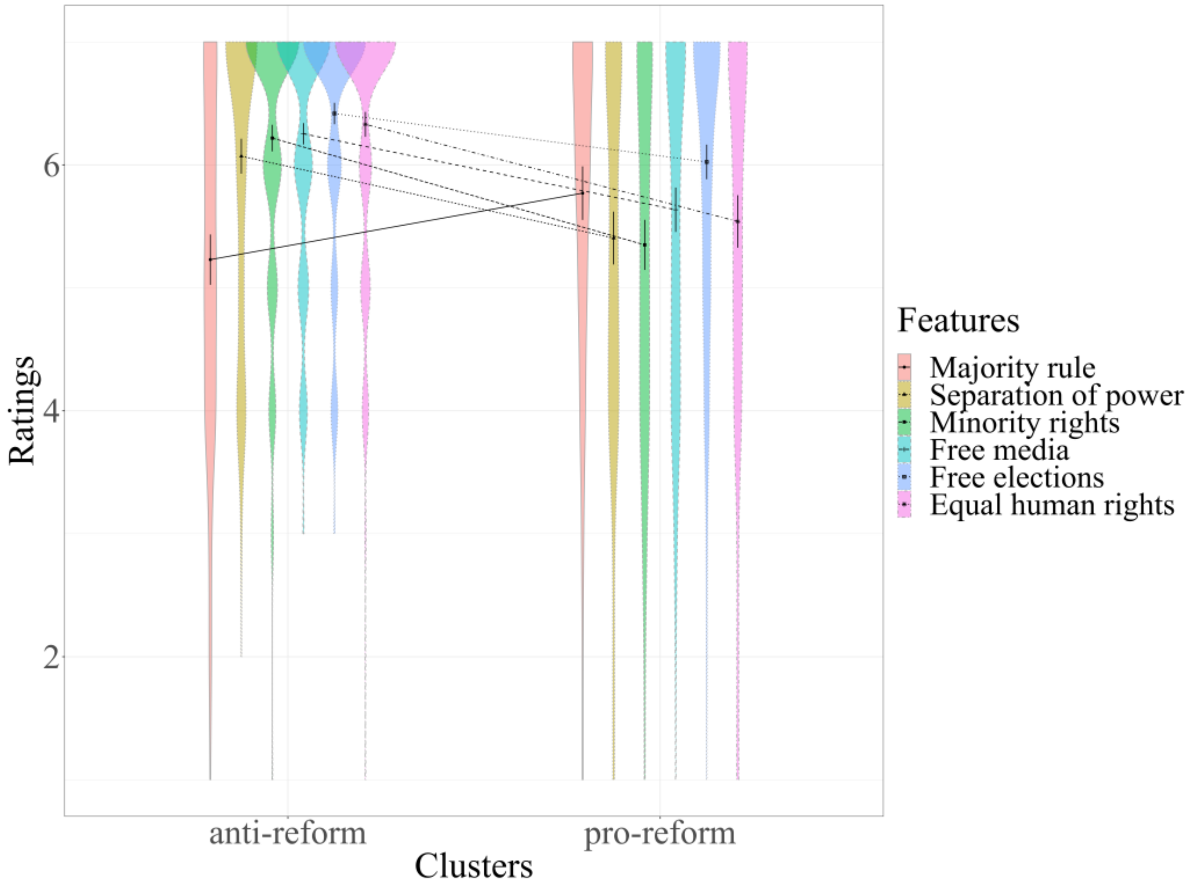

Ratings of importance of democracy features (1 = *not important at all*, 7 = *very important*) by cluster. Majority rule (light orange), separation of power (mustard), minority rights (green), free media (turquoise), free elections (purple), and equal human rights (pink). Both exclusion criteria applied (*N* = 300).
